# Automated lumbar spine segmentation in MRI using an enhanced U-Net with inception module and dual-output mechanism

**DOI:** 10.1038/s41598-025-20721-3

**Published:** 2025-11-10

**Authors:** Jaysel Theresa Silveira, Girisha S., Poornima Panduranga Kundapur

**Affiliations:** Department of Data Science and Computer Applications, Manipal Institute of Technology, Manipal Academy of Higher Education, Manipal, Karnataka 576104 India

**Keywords:** Semantic segmentation, Lumbar spine segmentation, U-Net, Inception module, Dual-output mechanism, Binary segmentation, Multiclass segmentation, Biomedical engineering, Medical research, Engineering

## Abstract

Accurate segmentation of spinal structures, including vertebrae, intervertebral discs (IVDs), and the spinal canal, is crucial for diagnosing lumbar spine disorders. Deep learning-based semantic segmentation has significantly improved accuracy in medical imaging. This study proposes an enhanced U-Net incorporating an Inception module for multi-scale feature extraction and a dual-output mechanism for improved training stability and feature refinement. The model is trained on the SPIDER lumbar spine MRI dataset and evaluated using Accuracy, Precision, Recall, F1-score, and mean Intersection over Union (mIoU). Comparative analysis with the baseline models—U-Net, ResUNet, Attention U-Net, and TransUNet—shows that the proposed model achieves superior segmentation accuracy, with improved boundary delineation and better handling of class imbalance. An evaluation of loss functions identified Dice loss as the most effective, enabling the model to achieve an mIoU of 0.8974, an accuracy of 0.9742, a precision of 0.9417, a recall of 0.9470, and an F1-score of 0.9444, outperforming all four baseline models. The Inception module enhances feature extraction at multiple scales, while the dual-output mechanism improves gradient flow and segmentation consistency. Initially focused on binary segmentation, the approach was extended to multiclass segmentation, enabling separate identification of vertebrae, IVDs, and the spinal canal. These enhancements offer a more precise and efficient solution for automated lumbar spine segmentation in MRI, thereby supporting enhanced diagnostic workflows in medical imaging.

## Introduction

Low back pain (LBP) is a significant global health concern, leading to activity limitations, work absences, and substantial economic burdens^1^. It is often associated with vertebral fractures, spinal misalignment, and degenerative disorders. Early and accurate diagnosis of spinal abnormalities is crucial for effective treatment and the prevention of severe complications such as chronic low back pain (cLBP)^2^.

The lumbar spine is made up of five vertebrae (L1–L5), which provide structural support to the upper body and facilitate movement in the lower back^3^. The lumbar cord, a section of the spinal cord passing through the lumbar vertebrae, transmits neural signals between the brain and lower body. Intervertebral discs (IVDs)–cartilaginous structures located between adjacent vertebrae–function as shock absorbers, reducing mechanical stress on the spine. The spinal canal, a bony passage that houses and protects the spinal cord and nerve roots, plays a vital role in maintaining neural function.

Degeneration of IVDs, commonly due to aging and reduced water content, compromises their shock-absorbing ability, contributing to LBP^4^. Conditions such as disc herniation, disc bulge, and lumbar spinal stenosis are major contributors to LBP. Herniation and bulging often lead to nerve compression, causing tingling and numbness in the legs^5,6^. Spinal stenosis, which involves the narrowing of the spinal canal, further complicates diagnosis due to the intricate soft tissue structures within the affected region^7^.

Medical imaging modalities such as magnetic resonance imaging (MRI) and computed tomography (CT) are essential for diagnosing spinal disorders. MRI is generally preferred for lumbar spine imaging due to its superior soft tissue contrast and absence of ionizing radiation^8^. However, the complexity of spinal anatomy and variations in image contrast present significant challenges for the accurate segmentation of spinal structures from medical images.

Accurate segmentation of vertebrae, IVDs, and the spinal canal is essential for identifying and delineating their boundaries in medical images, facilitating the assessment of spinal disorders, guiding treatment decisions, and enabling early intervention^9^. Simultaneous segmentation of multiple lumbar spine structures is more clinically relevant than segmenting each structure individually, as it helps identify pathological correlations and provides valuable insights for diagnosis and treatment. Traditionally, spinal image segmentation has been performed manually by radiologists, a time-consuming process prone to observer variability^10^. This manual approach requires expertise and effort to accurately annotate regions of interest (ROIs), making it inefficient for handling large datasets. Furthermore, the limited availability of publicly annotated medical datasets poses an additional challenge^11^.

With the growing demand for efficient spinal segmentation, automated techniques have gained popularity for detecting anatomical structures like vertebrae, IVDs, and the spinal canal, reducing radiologists’ workload. Among these, automatic semantic segmentation is widely used^12^, as it classifies each pixel based on its surrounding context using predefined categories established by radiologists^10^.

**Fig. 1:**
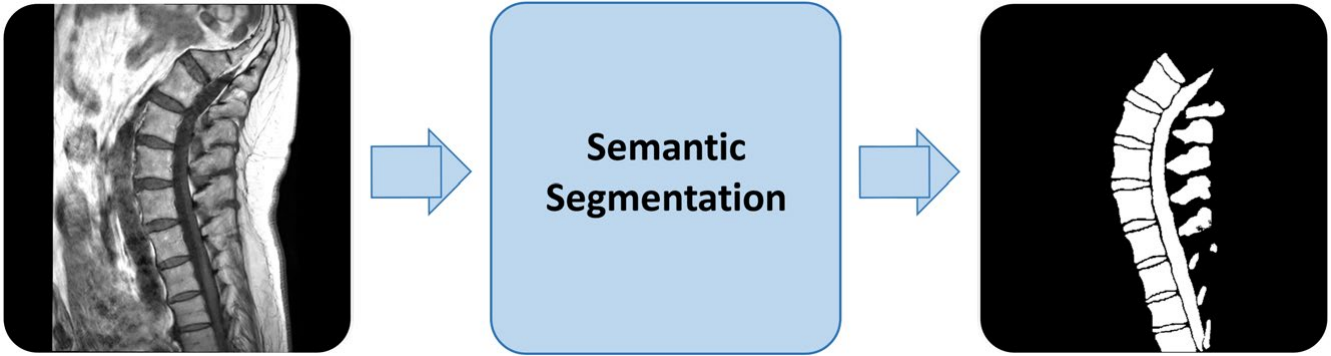
Overview of the semantic segmentation process applied to a sagittal T1-weighted spinal MRI image. Vertebrae, IVDs, and the spinal canal are identified as foreground, while all other regions are classified as background. The output is a binary segmentation map highlighting these anatomical structures.

Figure 1 illustrates how an input sagittal T1-weighted spinal MRI image is segmented into foreground and background regions through binary semantic segmentation. Here, key spinal structures–including vertebrae, IVDs, and the spinal canal–are identified as foreground, while the irrelevant areas are classified as background to produce the final segmentation output.

Classical image segmentation techniques, including thresholding, region growing, edge detection, and clustering, rely on low-level features like intensity and texture to partition a single image. While effective for basic tasks, these methods often require manual tuning and struggle with complex structures, limiting their ability to capture high-level semantic information. Originally designed for grayscale images, these methods face challenges in handling variations in scale, posture, and noise^13^. In lumbar spine imaging, their rigidity, especially in handling noise and grayscale variations, makes it challenging to accurately delineate vertebrae, IVDs, and shrinking spinal canal areas^14^, highlighting the need for more advanced, automated approaches.

Collaborative segmentation methods improve accuracy by identifying common objects across multiple images and leveraging shared features. However, they assume structural similarity across images, making them less effective in cases of significant variations in background, object appearance, or scale^15^. These limitations highlight the need for more robust approaches, such as deep learning-based segmentation, which can learn complex patterns from larger datasets.

Deep learning models demonstrate remarkable success in medical image segmentation^16^. Fully convolutional networks (FCNs), particularly U-Net, have gained popularity for their ability to perform pixel-wise classification with high precision. The encoder-decoder structure of U-Net ensures effective feature extraction, while skip connections help retain spatial details, addressing the vanishing gradient problem in deep architectures^17^. To improve segmentation accuracy, several U-Net variants have been proposed, incorporating architectural enhancements such as residual blocks, dense connections, multi-scale feature extraction, and attention mechanisms. Residual and dense blocks enhance feature reuse and gradient propagation, preventing the degradation of deep models^18,19^. Multi-scale processing is crucial for segmenting anatomical structures of varying shapes and sizes, as it enables the model to capture features at different resolutions^20,21^. Furthermore, attention mechanisms, such as channel and spatial attention modules, help the model focus on relevant features while suppressing background noise, improving boundary delineation, and mitigating class imbalance^22,23^.

Despite these advancements, accurate segmentation of spinal structures remains challenging due to overlapping tissue intensities, poor contrast, and anatomical variability across patients. Standard U-Net architectures may struggle to precisely capture both local details and broader contextual relationships, leading to suboptimal segmentation performance.

To address these challenges, this study proposes an enhanced U-Net-based model that integrates an Inception module to improve multi-scale feature extraction while maintaining spatial resolution through skip connections. Additionally, the model employs a dual-output mechanism, which enhances gradient flow during training and facilitates more stable convergence. By leveraging these architectural enhancements, the proposed model aims to improve the segmentation of spinal structures, particularly in handling anatomical variability, class imbalance, and boundary ambiguity.

The key contributions of this research include:

- Developing and evaluating a novel U-Net-based model designed to improve binary segmentation accuracy, where the foreground consists of spinal structures (vertebrae, IVDs, and the spinal canal) and the background represents all other regions.
- Comparing the performance of the proposed model against the baseline U-Net and its enhanced variants–ResUNet, Attention U-Net, and TransUNet–to demonstrate its effectiveness in capturing anatomical boundaries more precisely.
- Investigating the impact of training with different loss functions on segmentation performance.
- Extending the model to four-class multiclass segmentation for separate identification of vertebrae, IVDs, spinal canal, and the background.

The rest of this paper is structured as follows: Section Related work reviews previous research on traditional and deep learning-based methods for automatic spine segmentation. Section Methodology details the methodology, covering the proposed approach, data preprocessing steps, model design, and parameter optimization strategies. Section Results and discussion presents and discusses the results, including dataset details, performance metrics, evaluation of loss functions, ablation studies, comparative analysis, performance stability across multiple runs, model complexity and efficiency, Grad-CAM evaluation, and an extension to multiclass segmentation. Finally, Section Conclusion summarizes the key findings and outlines potential directions for future research.

## Related work

Medical image segmentation methods are broadly categorized into traditional and deep learning-based techniques.

### Traditional methods

Traditional segmentation techniques, also known as free estimation methods, do not rely on explicit models. Instead, they use image intensity, edges, or geometric properties to delineate anatomical structures. These methods include thresholding, region-based, clustering-based, and edge-based segmentation.

Thresholding classifies pixels based on intensity values for segmentation. Hoad et al.^24^ applied this method to segment lumbar discs from soft tissues in spine MRI images, facilitating computer-aided diagnosis. Similarly, Zhang et al.^25^ utilized a thresholding-based approach for CT vertebral image segmentation, isolating the bone region through image partitioning and refining segmentation using iterative correlation. While computationally efficient, thresholding struggles with poor contrast and overlapping intensities between different tissues.

Region-growing methods segment images by grouping neighboring pixels with similar intensities. Mastmeyer et al.^26^ used this technique to detect IVDs in spinal images, but it required manual parameter adjustments at multiple stages and user-defined seed points, limiting automation. Fu et al.^27^ introduced a 3D region-growing method for spinal canal segmentation in CT images, improving accuracy while preserving anatomical details, though it remained sensitive to noise and required careful seed point selection to prevent leakage into surrounding tissues.

Watershed segmentation, a region-based technique, treats an image as a topographic surface and identifies watershed lines to separate objects. Yao et al.^28^ combined watershed segmentation with directed graph search to locate vertebral body surfaces but reported leakage in ambiguous regions. Punarselvam et al.^29^ further demonstrated the challenge of over-segmentation in lumbar spine segmentation, requiring post-processing to refine boundaries. Silvoster et al.^30^ addressed this limitation by integrating edge detection with the Maximally Stable Extremal Regions (MSER) algorithm, highlighting the potential of combining MSER with image enhancement for improved segmentation accuracy.

Clustering-based segmentation groups pixels with similar characteristics to identify anatomical structures. Sabaghian et al.^31^ used K-means for 3D segmentation of the vertebral canal and thoracolumbar spinal cord from T2-weighted MRIs, outperforming manual methods but remaining sensitive to image quality. Athertya and Kumar^32^ applied Fuzzy C-Means (FCM) with morphological operations for vertebrae segmentation in T1-weighted MRIs, achieving better results than K-means and Otsu thresholding but still affected by noise. Ongoing research explores intuitionistic fuzzy clustering and feature extraction for improved spinal deformity classification.

Active Contour Models (ACMs), an edge-based segmentation approach, iteratively refine object boundaries by evolving curves toward high-gradient regions. Mahmoudi et al.^33^ extracted vertebral contours from X-ray images, refining them to achieve final segmentation. Athertya and Kumar^34^ enhanced ACMs by introducing a contour initialization technique for vertebra segmentation. Warrier and Viji^35^ applied ACMs to spinal canal segmentation, integrating K-means clustering to enhance boundary detection and improve segmentation accuracy.

Building on ACMs, Level Set Methods extend contour evolution to handle complex topological changes like merging or splitting boundaries. Liu et al.^36^ employed level sets for CT vertebral image segmentation, while Hille et al.^37^ utilized a hybridized level set approach. Boonyai et al.^38^ further enhanced the level set method by incorporating bone geometric analysis and polynomial estimation, improving vertebral segmentation and pose estimation, especially in low-radiation images. While these improvements enhance segmentation, accurately delineating complex anatomical environments and curved vertebral structures remains a challenge.

Despite advancements, traditional methods require manual tuning, struggle with noise, and lack robustness in handling complex anatomical structures. Deep learning overcomes these limitations with data-driven, automated, and more accurate segmentation.

### Deep learning-based methods

Deep learning-based segmentation techniques, or trainable methods, learn anatomical patterns from labeled data, assuming target structures follow repetitive geometric patterns that can be probabilistically modeled. Neural networks excel in spinal segmentation by learning hierarchical features, ensuring precise and robust results even in complex or low-contrast regions.

Early segmentation techniques focused on deep learning architectures like U-Net, SegNet, and AlexNet, as reviewed by J. Andrew et al.^39^. Their study highlighted U-Net’s superior accuracy, even with limited training data, making it a widely preferred approach. Building on this, Šušteršič et al.^5^ enhanced IVD segmentation by integrating contour-based cropping with U-Net, improving precision. Similarly, Lu et al.^40^ introduced a two-stage approach for lumbar vertebrae segmentation in CT images, where a U-Net-based localization network identified the lumbar spine region, followed by a 3D X-Unet model for precise segmentation, demonstrating the effectiveness of multi-stage pipelines.

Advancements in hybrid architectures emerged with Altun et al.^7^, who improved lumbar spinal stenosis segmentation by integrating ResNet, VGG16, and InceptionNet into U-Net, with ResUNet achieving the highest accuracy. Al-Kafri et al.^41^ further explored SegNet for lumbar spinal stenosis segmentation in MRI, comparing transfer learning with VGG16 (SegNet-TL) against training from scratch (SegNet-FS), where SegNet-TL outperformed due to transfer learning’s effectiveness.

A shift towards object detection and feature refinement was seen in the study by Pan et al.^6^, who employed Faster R-CNN for vertebral body localization and ResNet101 for IVD classification, incorporating a cost-sensitive CNN to address class imbalance. Deng et al.^42^ later proposed a U-Net and BiSeNet complementary model, integrating ResUNet for feature extraction, strip pooling for long-range dependencies, and attention refinement modules for better feature fusion, improving segmentation accuracy but still lacking spinal canal segmentation.

Further improvements in feature learning were introduced by Wang et al.^20^ with MLKCA-UNet, which integrates Multiscale Large-Kernel Convolution (MLKC) and CBAM attention into U-Net for better spatial feature extraction. In parallel, Han et al.^43^ developed Spine-GAN, a Recurrent Generative Adversarial Network (GAN) for semantic segmentation of spinal structures from MRIs. It incorporated atrous convolution, an autoencoder for fine-grained representation, LSTM to model spatial pathological relationships, and an auxiliary CNN for error correction, achieving high pixel accuracy and demonstrating clinical utility.

For handling noisy labels in medical imaging, Wang et al.^44^ proposed RAR-U-Net, which enhances feature learning with residual interconnections and attention mechanisms while dynamically filtering noisy labels. Cheng et al.^45^ introduced DAUNet++, an evolution of UNet++ with Residual blocks, Parallel and Series Spatial Modules for feature refinement, and CBAM for spatial and channel-wise enhancement, outperforming conventional models.

More recent advancements incorporate generative and transformer-based architectures. Liu et al.^46^ proposed a WGAN-based segmentation model integrating ResUNet and a Clustered Transformer Module (CTM) for vertebral MRI analysis. This approach transforms a GAN generator into a U-Net-based network with transformer architecture, improving structural accuracy and segmentation quality. The SPA-ResUNet proposed by Li et al.^47^ further enhanced segmentation by using ResUNet with Strip Pooling Attention (SPA) modules, refining accuracy in complex spinal structures.

Li et al.^48^ proposed ICUnet++, an improved Unet++ model for MR spine image segmentation, integrating an Inception structure for multi-scale feature extraction, CBAM for enhanced attention, and dense skip connections for refined learning. Finally, Zhang et al.^49^ introduced a two-stage segmentation approach using the 3D Swin Transformer. Their model, combining 3D Swin-YoloX for localization and 3D Swin-UNet for segmentation, effectively handles noise and artifacts, outperforming traditional convolution-based methods and marking a significant advancement in spinal segmentation.

#### Recent advances in deep medical image segmentation

While U-Net and its variants form the basis of deep medical image segmentation, recent models have introduced strategies like edge awareness, attention mechanisms, and automated configuration to improve performance. This section reviews key models and compares them with the proposed approach in terms of architectural innovations and performance characteristics.

BASNet, proposed by Qin et al.^50^, introduces a two-stage predict-refine framework comprising a deeply supervised encoder-decoder similar to the U-Net and a Residual Refinement Module (RRM). It employs a hybrid loss function that integrates Structural Similarity Index (SSIM), Binary Cross Entropy (BCE), and Intersection over Union (IoU) to enhance both regional accuracy and boundary precision. While effective in capturing fine structures and complex edges, the inclusion of dual encoder-decoder modules and multiple supervision outputs may increase model complexity and training overhead. In contrast, the proposed method adopts a simplified single-pass architecture that integrates an Inception module for efficient multi-scale feature extraction. Unlike BASNet, it does not perform explicit edge detection. Instead, the dual-output mechanism provides segmentation outputs at the bottleneck and final decoder layers, reinforcing semantic learning without adding computational burden. This enables competitive performance in binary and multi-class spine segmentation with fewer parameters and faster inference.

Chen et al.^51^ proposed TransUNet, a hybrid architecture that combines CNN-based feature extraction with Transformers to capture long-range dependencies. While effective for multi-organ segmentation due to its global context modeling, its patch-based encoding can lead to a loss of local spatial detail, necessitating cascaded upsampling to restore boundary precision. In contrast, the proposed model preserves fine spatial features through a compact encoder-decoder design with an integrated Inception module for multi-scale context. Additionally, the dual-output segmentation head enhances structural accuracy, particularly in small or overlapping anatomical regions.

Jha et al.^52^ introduced DoubleU-Net, a cascaded architecture of two sequential U-Nets, where the first leverages a pre-trained VGG-19 encoder and the second refines the output using Squeeze-and-Excitation (SE) and Atrous Spatial Pyramid Pooling (ASPP) blocks. This coarse-to-fine strategy enhances segmentation accuracy, particularly for small or flat polyps. However, the dual-stage design increases parameter count and training time. In contrast, the proposed model achieves comparable performance through a single-stage design using Inception-based downsampling and skip connections, eliminating the need for additional refinement modules.

Isensee et al.^53^ proposed nnU-Net, a self-configuring segmentation framework that automatically adapts preprocessing, network architecture, training, and post-processing based on dataset-specific characteristics. Its strength lies in robustness and generalization across a wide range of biomedical tasks without manual tuning. However, it relies heavily on heuristic rules and empirical design choices rather than fixed architectural priors, and its extensive pipeline configuration can increase training complexity and resource demand. In contrast, the proposed model leverages domain-specific design choices–such as Inception-based multi-scale fusion and dual-output supervision–to achieve high segmentation accuracy with a simpler, task-optimized architecture that is more interpretable and efficient in deployment scenarios.

#### Feature extraction strategies in spine imaging

Recent deep learning-based spine segmentation and classification models have adopted advanced feature extraction techniques to address anatomical complexity and variability. Common strategies include attention mechanisms, multi-scale feature fusion, and view-specific encoders.

The Automated Multi-scale Feature Fusion Network^54^ and its MRI-based variant (MSFF)^55^ introduced by Saeed et al. use hierarchical encoders with Feature Fusion Module (FFM), SE blocks, ASPP, and attention blocks such as Residual Convolution Block Attention Module (RCBAM), Local Position Residual Attention Block (LPRAB) and Residual Border Refinement Attention Block (RBRAB) to extract and refine multi-scale semantic features. While effective, the use of multiple specialized modules results in deeper architectures that may increase design and tuning complexity.

The 3D Multi-Feature Attention (3D MFA)^56^ model extracts features from sagittal, axial, and coronal views using separate MobileNetv3 encoders enhanced with Reverse CBAM and Feature Pyramid Pooling (FPP), which are fused into a 3D feature map. While MobileNetv3 is lightweight, the use of three independent encoders may still increase memory and compute demands compared to single-encoder designs. Similarly, 3D Mobile Residual U-Net (3D MRU-Net)^57^ uses MobileNetv2 with residual blocks to extract features from sagittal, axial, and coronal views using separate encoders. While lightweight, this view-specific strategy may introduce challenges in generalizing across modalities or anatomical variations.

Cascaded Hierarchical Atrous Spatial Pyramid Pooling Residual Attention U-Net (CHASPPRAU-Net)^58^ integrates cascaded spatial pyramid pooling and residual blocks in a U-Net encoder to extract features, while attention modules are applied in the decoder to enhance spatial focus. These additions increase the architectural depth and likely require module-specific design and tuning. Multi-scale Attention Feature Fusion MobileNetv3 (MAFMv3)^59^, introduced by Dastgir et al. and designed for spine lesion classification, combines MobileNetv3 with CBAM and ASPP to extract multi-scale features from raw, normalized, and histogram-equalized images, which are fused into a 3D feature map. While feature-rich, the model is not optimized for segmentation. Attention LinkNet-152^60^ uses modified EfficientNetB7 blocks with attention modules to extract features from sagittal, axial, and coronal CT views. These features are fused and passed to a LinkNet architecture that replaces the standard ResNet-34 encoder with a deeper ResNet-152 to enhance semantic representation. While this setup improves spatial focus and hierarchical depth, it increases architectural complexity and model size.

In contrast, the proposed model employs an Inception-based encoder for multi-scale feature extraction using parallel convolutions with varying receptive fields, enabling the capture of both fine and coarse spatial features. Unlike multi-view or attention-stacked architectures, it avoids the complexity of multiple encoders or deep attention pipelines. Designed to be lightweight and interpretable, the model incorporates skip connections and a dual-output segmentation mechanism to preserve spatial fidelity. Compared to the aforementioned works, it offers a favorable trade-off between computational efficiency and segmentation performance, making it suitable for clinical deployment.

An architectural comparison of recent segmentation models and the proposed model is presented in Table  1, highlighting architecture components, design highlights, and key observations related to complexity and performance.

**Table 1:** Architectural comparison of recent segmentation models and the proposed model.

Model	Architecture Components	Design Highlights	Key Observations
**BASNet** ^50^	U-Net + RRM	Two-stage predict-refine pipeline, deep supervision, hybrid loss (SSIM, BCE, IoU).	High model complexity and slower training due to multi-branch design.
**TransUNet** ^51^	ResNet + Transformer	Combines CNN-based feature extraction with Transformer-based global context modeling.	Loss of fine spatial detail due to patch embedding.
**DoubleU-Net** ^52^	VGG-19 + SE + ASPP	Cascaded U-Nets with attention and contextual refinement modules.	Higher parameter count and training time due to dual-stage architecture.
**nnU-Net** ^53^	Self-configuring U-Net	Automatic pipeline adaptation to dataset characteristics.	Resource-intensive; relies on heuristics for configuration and tuning.
**MSFF** ^54^ **/ MSFF-MRI** ^55^	Hierarchical Encoder + ASPP + SE + Attention	Multi-level fusion via FFM, RCBAM, LPRAB, RBRAB.	Deep architecture with complex integration; high tuning overhead.
**3D MFA** ^56^	MobileNetv3 + Reverse CBAM + FPP	Multi-view encoding (sagittal, axial, coronal) with attention-enhanced fusion.	Separate encoders for each view increase memory and computational cost.
**3D MRU-Net** ^57^	MobileNetv2 + Residual Blocks	Multi-view encoding using three residual encoders.	Lightweight per encoder, but reduced generalization across modalities.
**CHASPPRAU-Net** ^58^	U-Net + Residual + Attention	ASPP in encoder and attention in decoder for refined spatial focus.	Deep network may require extensive hyperparameter tuning.
**MAFMv3** ^59^	MobileNetv3 + CBAM + ASPP	Feature fusion from raw, normalized, and histogram-equalized images.	Optimized for classification; not directly suited for segmentation.
**Attention LinkNet-152** ^60^	EfficientNetB7 + ResNet-152 + LinkNet Decoder	Multi-view fusion with attention modules and deeper backbone.	Large model size and complexity increase training costs.
**Proposed Model**	Inception-based Encoder	Parallel convolutions with varying receptive fields, skip connections, dual-output segmentation head.	Lightweight; captures multi-scale context and improves gradient flow during training.

### Research gap

Despite significant advancements in spine segmentation, several challenges remain unaddressed. Many deep learning models, while achieving high accuracy, are computationally expensive, limiting real-time clinical applications. Generative and transformer-based models, though effective, require large datasets and high processing power, reducing scalability and accessibility. Another critical gap is the lack of contextual information, which is crucial for the accurate segmentation of complex spinal structures, particularly in low-contrast regions. Existing methods often focus on local features but fail to capture long-range dependencies, impacting segmentation precision and robustness.

While recent models achieve high accuracy, there is still room to improve the mIoU score. To address this, lightweight architectures are needed to retain high accuracy while decreasing computational overhead. Architectural modifications can help achieve this goal. In the context of this research, contextual information plays a key role in accurately segmenting the ROI, making it imperative to develop models that effectively capture spatial relationships while remaining computationally feasible. These gaps highlight the need for further research in designing segmentation models that are both accurate and lightweight, ensuring practical applicability in real-world medical imaging.

## Methodology

### Overview

The proposed model builds upon the standard U-Net by incorporating an Inception module to enhance feature extraction while preserving spatial details through skip connections. It follows an encoder-bottleneck-decoder structure, where the encoder extracts hierarchical features, the bottleneck refines feature representations, and the decoder reconstructs the segmentation map while maintaining spatial integrity. The model employs a dual-output mechanism, which improves gradient flow during training and provides deeper insights into the learning process. By effectively capturing both global and local contextual information, this architecture is designed to achieve more precise segmentation results.

### Data preprocessing

The data preprocessing pipeline ensures that the input images and ground truth masks are standardized and formatted appropriately for model training. The first step involves selecting only T1-weighted MRI images from the dataset due to their superior ability to visualize anatomical structures with high contrast.

Once the relevant images are identified, both the MRI scans and their corresponding ground truth masks are loaded. Since medical images are often three-dimensional, the central slice of each volume is extracted to maintain consistency across the dataset. The selected slices are then normalized to a range of [0, 255] to standardize intensity values, followed by conversion to an 8-bit format. The same preprocessing steps are applied to the ground truth masks to ensure alignment with the corresponding images.

To standardize the input dimensions, all images and masks are resized to 128$\times$128 pixels. The pixel values of the images are normalized to a [0,1] range to ensure stable training dynamics. The ground truth masks are then converted into a one-hot encoded format with two classes, distinguishing between background and foreground regions. This encoding enables pixel-wise classification, making it suitable for segmentation tasks.

In addition to the main segmentation ground truth masks, an auxiliary supervision signal is introduced by generating bottleneck ground truth masks, which are further downsampled to an 8x8 resolution to match the dimensions of the bottleneck output. This low-resolution supervision helps reinforce feature learning at multiple scales.

Finally, the preprocessed images and their corresponding masks are stored as NumPy arrays for efficient loading during training. This structured preprocessing approach ensures that all inputs are standardized, aligned, and formatted optimally, contributing to improved segmentation performance.

### Model design

**Fig. 2:**
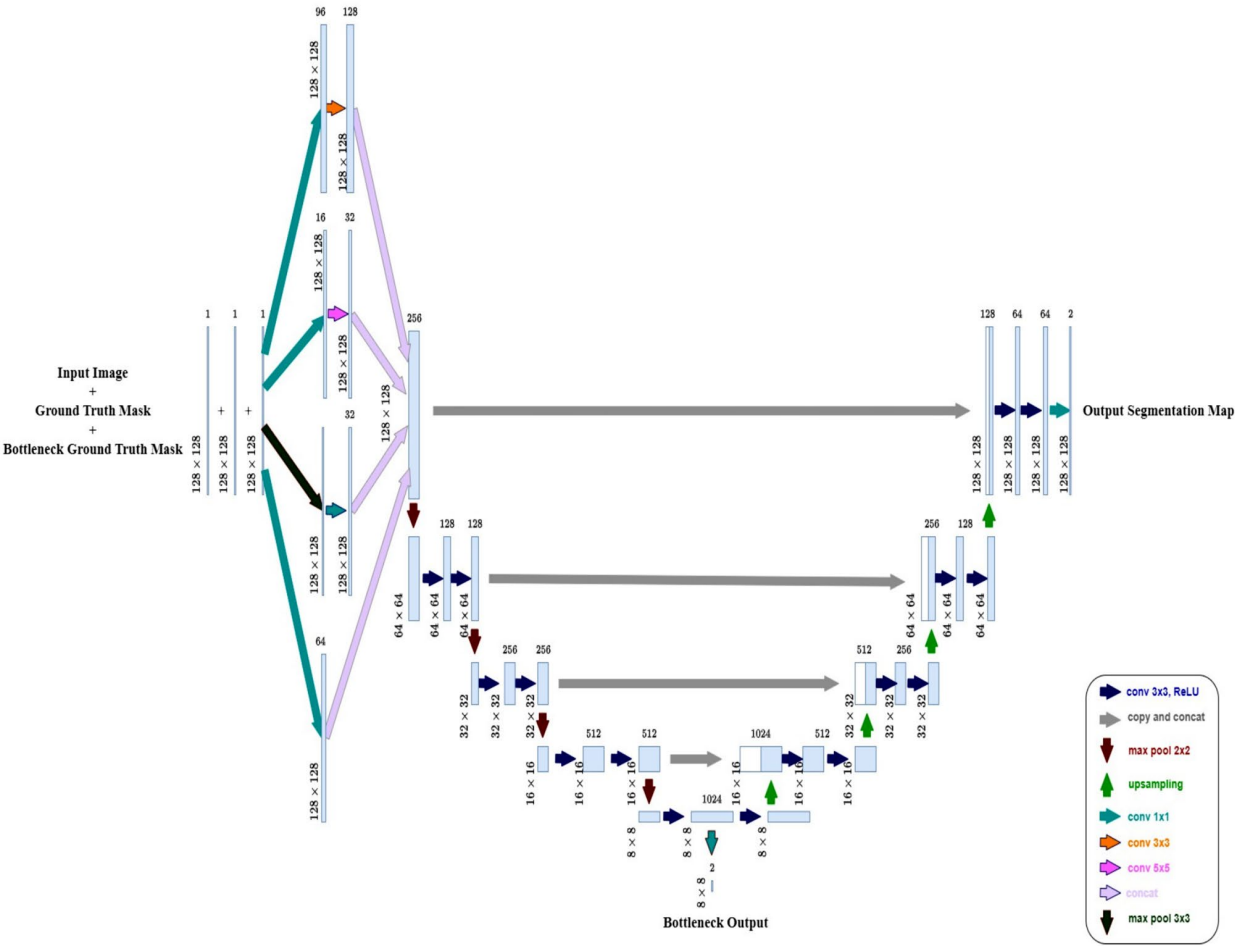
Architecture of the proposed model, which extends U-Net by incorporating an Inception module in the first encoder block for multi-scale feature extraction and a dual-output mechanism that provides segmentation outputs at both the bottleneck and final decoder layers.

Figure 2 illustrates the proposed model architecture, which is based on the U-Net framework with a key modification in the encoder, where the first encoder block is replaced with an Inception module to enhance feature extraction by incorporating multiple convolutional pathways, allowing the network to capture both fine and coarse details. Specifically, it consists of four parallel branches: a 1$\times$1 convolution with 64 filters for local feature extraction, a 1$\times$1 convolution followed by a 3$\times$3 convolution with 96 and 128 filters, respectively, for medium-scale feature extraction, a 1$\times$1 convolution followed by a 5$\times$5 convolution with 16 and 32 filters to capture larger patterns, and a 3$\times$3 max pooling layer followed by a 1$\times$1 convolution with 32 filters to retain spatial information while reducing computational complexity. The outputs from all branches are concatenated to form a rich multi-scale representation, which strengthens the encoder’s ability to extract diverse features at different receptive fields.

Following the Inception-enhanced first block, the encoder consists of three additional blocks, each containing two sequential 3$\times$3 convolutional layers with ReLU activation and Batch Normalization, followed by a 2$\times$2 max pooling layer for spatial downsampling. At each stage, the number of filters doubles (128, 256, and 512), ensuring the network captures increasingly complex features. To prevent overfitting, dropout layers (0.5) are incorporated in the deeper layers of the encoder.

At the bottleneck, the network consists of two 3$\times$3 convolutional layers with 1024 filters, each followed by ReLU activation, Batch Normalization, and Dropout (0.5) to improve generalization. Additionally, an intermediate output, referred to as the bottleneck output, is extracted at this stage using a 1$\times$1 convolutional layer with 2 filters and a Softmax activation. This auxiliary output captures the deepest feature representations and serves as an additional supervision signal during training.

The decoder mirrors the encoder, with upsampling (2$\times$2) operations at each stage. The upsampled feature maps are concatenated with their corresponding encoder outputs through skip connections, ensuring that high-resolution spatial details are preserved. Each upsampling stage is followed by two 3$\times$3 convolutional layers with Batch Normalization to refine the feature maps. The final segmentation output is obtained through a 1$\times$1 convolutional layer with 2 filters and a Softmax activation, producing a pixel-wise classification map. A dual-output strategy is employed, generating both the final segmentation output and an intermediate bottleneck output, which enhances gradient flow during training and aids in feature refinement.

Integrating an Inception module at the first encoder block enables the network to capture multi-scale spatial information from the initial stage, enhancing feature representation for improved segmentation accuracy. The addition of a bottleneck output provides auxiliary supervision, refining feature learning while aiding gradient flow. These modifications strengthen the model’s performance while retaining the core advantages of the U-Net architecture.

### Parameter optimization

#### Loss function

The model is trained with Binary Cross-Entropy (BCE) loss, Dice loss, Focal loss, and a combined BCE-Dice loss to identify the most effective loss function for segmentation performance. BCE loss ensures pixel-wise classification, Dice loss emphasizes class imbalance handling by maximizing the overlap between ground truth and predicted masks and Focal loss addresses hard-to-classify regions by focusing more on challenging pixels. The combined BCE-Dice loss leverages the strengths of both BCE and Dice loss to enhance segmentation accuracy. The best-performing loss function was selected based on the model’s performance on the test set.

The BCE loss is defined as:

1 $$\begin{aligned} \mathscr {L}_{BCE} = - \frac{1}{N} \sum _{i=1}^{N} \left[ y_i \log (\hat{y}_i) + (1 - y_i) \log (1 - \hat{y}_i) \right] \end{aligned}$$

Where $y_i$ represents the true class label, $\hat{y}_i$ denotes the predicted probability of a pixel belonging to the foreground class, and $N$ is the total number of pixels in the image.

The Dice loss is given by:

2 $$\begin{aligned} \mathscr {L}_{Dice} = 1 - \frac{2 \sum _{i=1}^{N} y_i \hat{y}_i}{\sum _{i=1}^{N} y_i + \sum _{i=1}^{N} \hat{y}_i} \end{aligned}$$

Where $y_i$ represents the true class label, $\hat{y}_i$ denotes the predicted probability of a pixel belonging to the foreground class, and $N$ is the total number of pixels in the image.

The Focal loss modifies BCE by adding a scaling factor to focus on hard examples:

3 $$\begin{aligned} \mathscr {L}_{Focal} = - \frac{1}{N} \sum _{i=1}^{N} \alpha _i (1 - p_i)^\gamma \log (p_i) \end{aligned}$$

Where $p_i$ is defined as:

4 $$\begin{aligned} p_i = {\left\{ \begin{array}{ll} \hat{y}_i, & \text {if } y_i = 1 \\ 1 - \hat{y}_i, & \text {if } y_i = 0 \end{array}\right. } \end{aligned}$$

Here, $y_i$ represents the true class label, $\hat{y}_i$ denotes the predicted probability of a pixel belonging to the foreground class, and $N$ is the total number of pixels in the image. The factor $\alpha _i$ balances class imbalance, and $\gamma$ controls the focus on hard-to-classify samples by down-weighting easy examples. $\alpha$ is set to 0.5 to give equal importance to both classes and $\gamma$ is set to 2.0 to enhance the focus on hard-to-classify samples.

The combined BCE-Dice loss is formulated as:

5 $$\begin{aligned} \mathscr {L}_{BCE-Dice} = \lambda _1 \mathscr {L}_{BCE} + \lambda _2 \mathscr {L}_{Dice} \end{aligned}$$

Where $\lambda _1$ and $\lambda _2$ are weighting factors controlling the contribution of each loss component. Both weights are set to 1, ensuring equal contribution from BCE loss and Dice loss in the total loss calculation.

#### Optimizer and learning rate

The Adam optimizer is used for its adaptive learning rate and efficient handling of non-stationary objectives. By combining momentum and RMSProp, it ensures faster convergence and stability. A learning rate of 0.0001 is chosen to prevent large weight updates, allowing gradual parameter refinement, improved generalization, and reduced risk of overshooting the optimal solution.

#### Batch size and number of epochs

A batch size of 2 is used to balance memory constraints and training stability. Given the high-resolution nature of medical images, a smaller batch size accommodates computational demands while ensuring effective gradient updates. The model is trained for 50 epochs, allowing sufficient learning while minimizing the risk of overfitting.

## Results and discussion

### Dataset

This research utilized the publicly available multicenter lumbar spine MRI SPIDER dataset^61^, an open-source dataset accessible at https://spider.grand-challenge.org/data/. The dataset comprises imaging studies from 257 patients with a history of LBP, with each study including up to three MRI series, totaling 544 series. The publicly released portion consists of 218 patient studies with 447 MRI series.

The dataset contains sagittal T1- and T2-weighted MRI scans with resolutions ranging from 3.3 $\times$ 0.33 $\times$ 0.33 mm to 4.8 $\times$ 0.90 $\times$ 0.90 mm. Additionally, sagittal T2 SPACE sequence images provide near-isotropic spatial resolution with a voxel size of 0.90 $\times$ 0.47 $\times$ 0.47 mm.

For this study, a total of 196 sagittal T1-weighted MRI images were used. T1-weighted images were selected for their superior ability to visualize anatomical structures, including vertebrae, IVDs, and the spinal canal, as well as to assess structural abnormalities. These images highlight fatty tissue by enhancing its signal while reducing the signal from water, creating high contrast between bone, soft tissue, and fat^62^. This contrast makes T1-weighted MRI particularly effective for segmentation tasks requiring precise boundary delineation.

80% of the total images were allocated for training, with 20% of that reserved for validation during each experiment. The remaining 20% of the dataset served as the test set. Cross-validation was not employed in this study; however, a consistent train-test split was used across all experiments to ensure fair and reproducible comparisons. To assess robustness, multiple independent runs were conducted for the proposed model using different random seeds, with the validation set re-sampled from the training data in each run.

The dataset provides ground truth masks that assign each pixel to one of multiple classes. A medical trainee performed the segmentation under the supervision of an experienced musculoskeletal radiologist and a medical imaging expert. To aid annotation, a small portion of the dataset was initially used to train an automatic segmentation model. The predicted segmentation outputs were then reviewed and corrected manually before being incrementally added to the training set until all images were annotated.

**Fig. 3:**
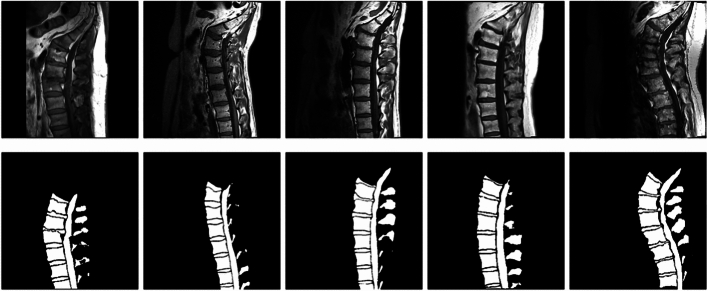
Sagittal spinal MRI images alongside their corresponding ground truth masks from the SPIDER dataset^61^, showing annotated anatomical structures.

To align with the study’s objectives, the ground truth masks were preprocessed to simplify segmentation into two categories: background and foreground. All visible vertebrae, IVDs, and the spinal canal were grouped into a single foreground class. Figure 3 presents sagittal spinal MRI images alongside their corresponding ground truth masks, illustrating the final annotations.

These structured annotations are crucial for supervised learning, enabling deep-learning models to learn precise anatomical delineations. The binary segmentation approach focuses on distinguishing spinal structures from the background, aiding automated spinal pathology detection, treatment planning, and clinical decision-making.

### Performance metrics

In this study, the U-Net^17^ architecture was used as the baseline model for semantic segmentation of spinal structures, with ResUNet^63^, Attention U-Net^64^, and TransUNet^51^ explored as alternative baseline models. ResUNet integrates residual blocks to improve gradient flow and convergence, Attention U-Net uses attention gates to enhance focus on relevant spinal regions and suppress less informative features, and TransUNet leverages transformers and self-attention mechanisms to capture long-range dependencies in medical images, thereby improving spinal structure segmentation.

The four baseline models were trained with four different loss functions–BCE, Dice, Focal, and a combined BCE-Dice–and assessed based on key performance metrics: Accuracy, Precision, Recall, F1-score, and mIoU. Accuracy reflects the overall proportion of correctly classified pixels, Precision measures the model’s ability to avoid false positives in identifying spinal structures, and Recall captures its ability to detect all relevant regions. The F1-score balances Precision and Recall, while mIoU quantifies the overlap between actual and predicted regions, providing a robust measure of segmentation performance across all classes.

Among all the baseline models, U-Net trained with BCE loss achieved the highest mIoU (0.8874), accuracy (0.9718), precision (0.9402), and F1-score (0.9384), along with a notable recall (0.9366). ResUNet, Attention UNet, and TransUNet demonstrated slightly lower performance across most metrics, indicating that the standard U-Net structure was more effective for lumbar spine segmentation. Based on these results, U-Net was selected as the foundation for the proposed model, which was further enhanced with bottleneck loss and an Inception block to improve feature extraction and segmentation accuracy. The proposed model significantly outperformed the standard U-Net and its variants, achieving the highest mIoU of 0.8974 when trained with Dice loss. Additionally, training with a combined BCE-Dice loss function resulted in the second-highest mIoU of 0.8928. These findings suggest that multi-scale feature extraction (via the Inception block) and improved gradient flow (via bottleneck loss) enhanced the model’s segmentation capabilities.

### Evaluation of loss functions

The baseline models and the proposed model were evaluated using different loss functions, with performance metrics summarized in Table 2.

**Table 2:** Comparison of segmentation performance for baseline and proposed models trained with four different loss functions. The best results are indicated in bold.

Model	Loss Function	mIoU	Accuracy	Precision	Recall	F1-score
U-Net	BCE	0.8874	0.9718	0.9402	0.9366	0.9384
	Dice	0.8822	0.9694	0.9242	**0.9474**	0.9353
	Focal	0.8788	0.9694	0.9347	0.9318	0.9332
	BCE-Dice	0.8818	0.9702	0.9359	0.9342	0.9351
ResUNet	BCE	0.8797	0.9692	0.9295	0.9383	0.9338
	Dice	0.8803	0.9696	0.9325	0.9358	0.9341
	Focal	0.8711	0.9667	0.9232	0.9342	0.9286
	BCE-Dice	0.8791	0.9693	0.9331	0.9338	0.9334
Attention U-Net	BCE	0.8739	0.9679	0.9305	0.9300	0.9303
	Dice	0.8798	0.9690	0.9255	0.9428	0.9339
	Focal	0.8707	0.9667	0.9240	0.9327	0.9283
	BCE-Dice	0.8796	0.9696	0.9363	0.9311	0.9337
TransUNet	BCE	0.8842	0.9709	0.9381	0.9349	0.9365
	Dice	0.8852	0.9706	0.9307	0.9438	0.9371
	Focal	0.8781	0.9689	0.9311	0.9346	0.9328
	BCE-Dice	0.8844	0.9707	0.9350	0.9383	0.9366
**Proposed Model**	BCE	0.8849	0.9715	**0.9450**	0.9291	0.9369
	**Dice**	**0.8974**	**0.9742**	0.9417	0.9470	**0.9444**
	Focal	0.8716	0.9677	0.9350	0.9228	0.9288
	BCE-Dice	0.8928	0.9733	0.9448	0.9384	0.9416

**Fig. 4:**
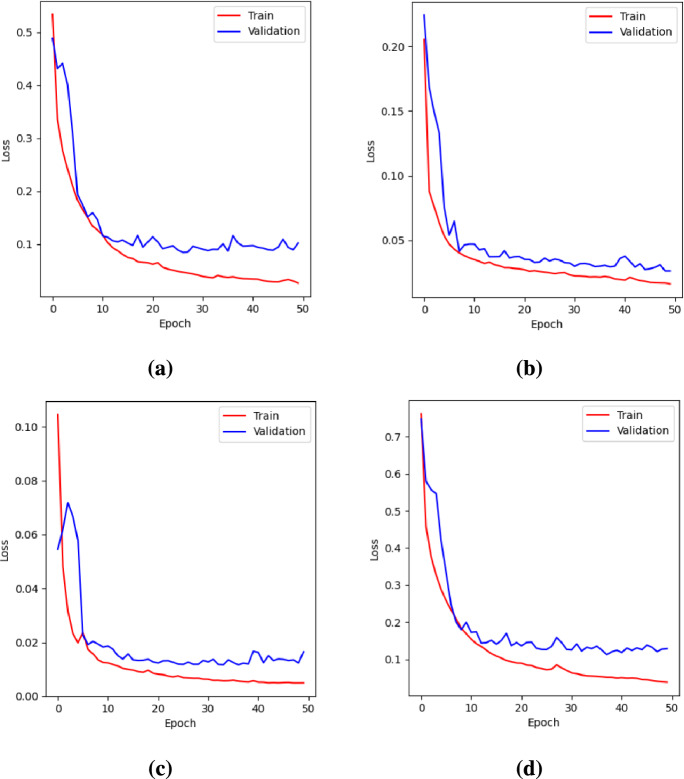
Loss curves for the proposed model using different loss functions during training and validation. (**a**) BCE loss. (**b**) Dice loss. (**c**) Focal loss. (**d**) BCE-Dice loss.

The proposed model was trained using BCE, Dice, Focal, and a combined BCE-Dice loss to assess their impact on lumbar spine segmentation. Among these, Dice loss yielded the best performance, achieving the highest mIoU (0.8974), accuracy (0.9742), and F1-score (0.9444), along with strong precision (0.9417) and recall (0.9470). This indicates that Dice loss was the most effective at optimizing the overlap between ground truth and predicted masks, thus making it well-suited for this task.

The combined BCE-Dice loss provided the second-best performance, with an mIoU of 0.8928. It effectively balanced precision and recall, making it a strong alternative to Dice loss alone.

While BCE loss underperformed in mIoU compared to Dice-based losses, it achieved the highest precision (0.9450) among all loss functions, suggesting that it was more conservative in making positive predictions. This could be beneficial in applications where accurately identifying spinal structures without overestimation is crucial.

Conversely, Focal loss exhibited the lowest performance across most metrics. Since the dataset did not exhibit severe class imbalance, Focal loss may have over-penalized easily classified pixels, leading to suboptimal segmentation results.

A similar trend was observed across all four baseline models (U-Net, ResUNet, Attention U-Net, and TransUNet), where Dice loss consistently provided the best segmentation quality in most cases, followed by BCE-Dice, BCE, and then Focal loss. These findings highlight Dice-based losses as the most effective for lumbar spine segmentation, while BCE loss remains a viable alternative for applications requiring higher precision.

Figure 4 presents the training and validation loss curves for the proposed model with different loss functions.

### Ablation studies

To assess the contribution of individual architectural components, ablation studies were conducted by comparing the baseline U-Net with variants incorporating either the Inception module or the dual-output mechanism, as well as the full proposed model integrating both components.

As shown in Table 3, incorporating the Inception module improved mIoU from 0.8822 to 0.8941, highlighting its effectiveness in multi-scale feature extraction through parallel convolutional paths with varied receptive fields. In contrast, using only the dual-output mechanism slightly reduced mIoU to 0.8716, indicating limited benefit when auxiliary supervision is applied without enhanced feature representations. The full proposed model, combining both components, achieved the highest mIoU of 0.8974, suggesting that the dual-output mechanism is most effective when supported by richer feature encoding from the Inception module.

**Table 3:** Comparison of segmentation performance across model variants: baseline U-Net, versions with the Inception module or dual-output mechanism individually, and the full proposed model integrating both components. All models were trained using Dice loss. The best results are indicated in bold.

Model	mIoU	Accuracy	Precision	Recall	F1-score
U-Net	0.8822	0.9694	0.9242	**0.9474**	0.9353
U-Net + Inception module	0.8941	0.9732	0.9382	0.9468	0.9424
U-Net + Dual-Output mechanism	0.8716	0.9669	0.9240	0.9340	0.9289
**Proposed Model**	**0.8974**	**0.9742**	**0.9417**	0.9470	**0.9444**

Further ablation studies were conducted to evaluate the impact of attention mechanisms on segmentation performance. The baseline models–U-Net and ResUNet–along with the proposed model were tested with attention mechanisms, using the best-performing loss function for each based on mIoU. Table 4 presents the results of integrating different attention mechanisms into the skip connections of each model to assess their effect.

**Table 4:** Comparison of segmentation models with and without attention mechanisms. All models were trained using their best-performing loss functions. The best results are indicated in bold.

Model	Loss Function	mIoU	Accuracy	Precision	Recall	F1-score
U-Net	BCE	0.8874	0.9718	0.9402	0.9366	0.9384
U-Net + SAM	BCE	0.8797	0.9694	0.9324	0.9352	0.9338
U-Net + CBAM	BCE	0.8792	0.9698	0.9398	0.9274	0.9335
ResUNet	Dice	0.8803	0.9696	0.9325	0.9358	0.9341
ResUNet + SAM	BCE-Dice	0.8785	0.9691	0.9323	0.9339	0.9331
ResUNet + CBAM	Dice	0.8739	0.9672	0.9213	0.9399	0.9303
**Proposed Model**	**Dice**	**0.8974**	**0.9742**	0.9417	**0.9470**	**0.9444**
Proposed Model + SE block	BCE-Dice	0.8893	0.9726	**0.9474**	0.9321	0.9395
Proposed Model + SAM	BCE-Dice	0.8865	0.9716	0.9416	0.9342	0.9379
Proposed Model + CBAM	Dice	0.8816	0.9698	0.9312	0.9388	0.9349

Adding the Spatial Attention Module (SAM) and Convolutional Block Attention Module (CBAM)^65^ to U-Net and ResUNet in the skip connections did not lead to significant performance improvements in lumbar spine segmentation. However, SAM consistently outperformed CBAM, likely because spatial attention effectively enhances focus on relevant anatomical structures, whereas CBAM’s combined spatial and channel attention mechanisms may have introduced redundancy.

Similarly, incorporating SAM and CBAM into the proposed model resulted in a decline in performance compared to the original proposed model. The SAM-enhanced proposed model achieved an mIoU of 0.8865, while the CBAM-enhanced variant performed slightly worse (mIoU: 0.8816). This suggests that while attention mechanisms can refine feature selection, they did not significantly enhance segmentation for this task.

Additionally, integrating a Squeeze-and-Excitation (SE)^66^ block in the skip connections for channel-wise attention resulted in a slightly lower mIoU of 0.8893, compared to the original proposed model. However, this configuration achieved the highest precision (0.9474) among all models. This suggests that while SE blocks enhance feature recalibration, their overall impact on segmentation performance was limited, possibly because the Inception block in the proposed model already captures key features effectively.

These findings suggest that the Inception module already captures spatial and multi-scale contextual features, reducing the benefit of additional attention mechanisms. Adding attention blocks increased the parameter count and complexity. As a result, attention modules did not provide complementary gains in this setting, highlighting the effectiveness and efficiency of the proposed model.

To evaluate the effect of data augmentation, the proposed model was trained using standard techniques, including rotations, width and height shifts, zooming, and horizontal flips, with nearest-neighbor filling for empty regions. Training used Dice loss, the best-performing loss function based on mIoU.

**Table 5:** Comparison of the proposed model’s segmentation performance with and without data augmentation. All models were trained using Dice loss. The best results are indicated in bold.

Model	mIoU	Accuracy	Precision	Recall	F1-score
**Proposed Model**	**0.8974**	**0.9742**	**0.9417**	**0.9470**	**0.9444**
Proposed Model + Data Augmentation	0.8667	0.9647	0.9127	0.9404	0.9259

As reported in Table 5, incorporating augmentation led to a decline in segmentation performance, with an mIoU of 0.8667 compared to 0.8974 without augmentation. This suggests that augmentation introduced variability that disrupted the spatial consistency of anatomical structures, particularly given the natural alignment in sagittal T1-weighted MRI scans. Consequently, the proposed model trained without augmentation yielded better performance, indicating that the dataset already contained sufficient diversity and that structural distortions introduced by augmentation were detrimental to learning.

### Comparative analysis

Table 6 presents a comparative assessment of the proposed model against the four baseline architectures for binary spine segmentation. All models were trained using the best-performing Dice loss. The results demonstrate that the proposed model consistently outperformed U-Net, ResUNet, Attention U-Net, and TransUNet across most performance metrics. Specifically, the proposed model achieved the highest mIoU (0.8974), accuracy (0.9742), precision (0.9417), and F1-score (0.9444), along with a strong recall (0.9470).

**Table 6:** Comparative analysis of segmentation performance across different models trained with Dice loss. The best results are indicated in bold.

Model	mIoU	Accuracy	Precision	Recall	F1-score
U-Net	0.8822	0.9694	0.9242	**0.9474**	0.9353
ResUNet	0.8803	0.9696	0.9325	0.9358	0.9341
Attention U-Net	0.8798	0.9690	0.9255	0.9428	0.9339
TransUNet	0.8852	0.9706	0.9307	0.9438	0.9371
**Proposed Model**	**0.8974**	**0.9742**	**0.9417**	0.9470	**0.9444**

Compared to the baseline U-Net, which achieved an mIoU of 0.8822, accuracy of 0.9694, precision of 0.9242, recall of 0.9474, and F1-score of 0.9353, the proposed model demonstrated an improvement of 1.72% in mIoU, 0.48% in accuracy, 1.75% in precision, and 0.97% in F1-score, while maintaining a comparable recall. These enhancements highlight the proposed model’s superior segmentation capabilities, particularly in distinguishing lumbar spine structures from the background. The increase in mIoU reflects better overall segmentation quality, while higher accuracy confirms improved classification of structures and backgrounds. The notable rise in precision indicates reduced false positives and sharper boundary delineation, which is essential for accurate vertebral segmentation. Despite a marginal decrease in recall, the overall improvement in the F1-score reinforces the model’s ability to effectively balance precision and recall, highlighting its reliability and balanced performance in lumbar spine segmentation.

**Fig. 5:**
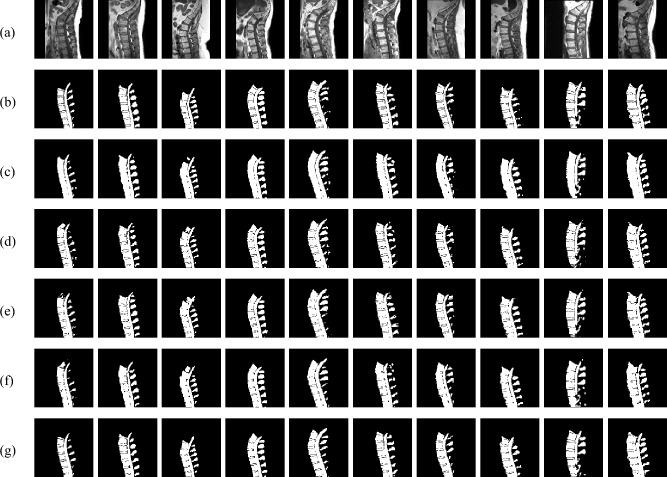
Test images with corresponding ground truth masks, predictions from four baseline models, and proposed model predictions. (**a**) Input Images. (**b**) Ground Truth Masks. (**c**) U-Net. (**d**) ResUNet. (**e**) Attention U-Net. (**f**) TransUNet. (**g**) Proposed Model.

The quantitative improvements are further reinforced by qualitative results in Fig. 5, which showcases test images, ground truth masks, predictions from four baseline models, and proposed model predictions, illustrating the latter’s enhanced segmentation performance.

The proposed model demonstrates superior boundary delineation and reduced misclassifications compared to all baseline models, ensuring better preservation of critical anatomical details. This improvement is particularly important for lumbar spine segmentation, where accurate localization of structural boundaries is essential. The results highlight the advantages of the proposed model’s enhanced architecture, reinforcing its effectiveness over traditional U-Net variants in accurately segmenting spinal structures.

To further assess the boundary accuracy of the proposed model, we computed two contour-based metrics: Hausdorff Distance (HD) and Average Symmetric Surface Distance (ASSD), both widely used in medical image segmentation to quantify the boundary alignment between predicted and ground truth masks. HD measures the maximum boundary deviation, while ASSD captures the average surface-to-surface distance. For our proposed model, the HD and ASSD were 8.5621 and 0.7059 pixels, respectively. Based on standard interpretation ranges–where HD values below 10 pixels and ASSD values below 1 pixel indicate good boundary agreement–these results suggest that our model produces anatomically accurate and spatially consistent segmentations with minimal surface discrepancy.

### Performance stability across multiple runs

To assess the robustness of the proposed model, 10 independent training runs were conducted using different random seeds with a fixed data split. Table 7 reports the segmentation metrics for each run along with the mean ± standard deviation, providing a comprehensive view of both performance and variability. The mean reflects average segmentation accuracy, while the standard deviation captures the model’s sensitivity to initialization and training conditions.

**Table 7:** Segmentation performance of the proposed model across 10 independent runs. Values shown in bold represent the Mean ± Standard Deviation.

Run	mIoU	Accuracy	Precision	Recall	F1-score
1	0.8891	0.9722	0.9415	0.9373	0.9394
2	0.8905	0.9726	0.9413	0.9391	0.9402
3	0.8906	0.9728	0.9457	0.9350	0.9403
4	0.8880	0.9724	0.9480	0.9300	0.9387
5	0.8869	0.9721	0.9487	0.9281	0.9381
6	0.8895	0.9729	0.9515	0.9285	0.9396
7	0.8899	0.9728	0.9478	0.9323	0.9399
8	0.8896	0.9724	0.9425	0.9370	0.9397
9	0.8875	0.9722	0.9479	0.9295	0.9384
10	0.8895	0.9721	0.9381	0.9413	0.9397
**Mean** ± **Std. Dev.**	**0.8892** ± **0.0012**	**0.9725** ± **0.0003**	**0.9453** ± **0.0041**	**0.9339** ± **0.0046**	**0.9394** ± **0.0008**

The findings demonstrate the proposed model’s consistent performance across 10 independent runs. It achieved an average mIoU of 0.8892 ± 0.0012, accuracy of 0.9725 ± 0.0003, precision of 0.9453 ± 0.0041, recall of 0.9339 ± 0.0046, and F1-score of 0.9394 ± 0.0008. The low standard deviations indicate minimal sensitivity to initialization and training variation, highlighting the model’s robustness. These results confirm that the proposed architecture is both accurate and stable, making it suitable for clinical deployment.

### Model complexity and efficiency

The computational efficiency of the proposed model was assessed by comparing its total, trainable, and non-trainable parameter counts with those of the baseline architectures. As presented in Table 8, the proposed model consisted of 31.47 million parameters, with 31.46 million trainable parameters and a minimal number of non-trainable parameters (12,256).

**Table 8:** Comparison of model complexity in terms of total, trainable, and non-trainable parameter counts. The proposed model’s values are indicated in bold.

Model	Total Params	Trainable Params	Non-trainable Params
U-Net	31,050,114	31,040,386	9,728
ResUNet	32,461,634	32,443,970	17,664
Attention U-Net	31,753,414	31,741,638	11,776
TransUNet	23,979,010	23,969,282	9,728
**Proposed Model**	**31,475,396**	**31,463,140**	**12,256**

Compared to the baseline U-Net (31.05 million parameters), the proposed model introduced a slight increase in complexity while achieving significantly better segmentation performance. ResUNet (32.46 million parameters) incurred a higher computational cost yet did not outperform the proposed model in segmentation accuracy. Attention U-Net (31.75 million parameters) had more parameters than the proposed model, while TransUNet (23.97 million parameters) had the fewest. However, neither achieved segmentation accuracy comparable to the proposed model.

Despite having a slightly higher parameter count than U-Net and TransUNet, the proposed model achieved a favorable balance between computational efficiency and segmentation accuracy, demonstrating its ability to improve performance without incurring excessive computational overhead.

### Grad-CAM evaluation

To enhance the interpretability of the proposed model’s predictions, Grad-CAM was applied alongside segmentation. This technique is particularly valuable in spinal MRI analysis as it helps radiologists and medical professionals understand which anatomical structures influence the model’s decisions. Grad-CAM generates visual heatmaps that highlight the most critical regions in the input MRI scans, providing insights into the model’s focus during segmentation^67,68^. These heatmaps validate the model’s attention to key spinal structures, including vertebrae, IVDs, and the spinal canal, improving the transparency and reliability of automated segmentation.

**Fig. 6:**
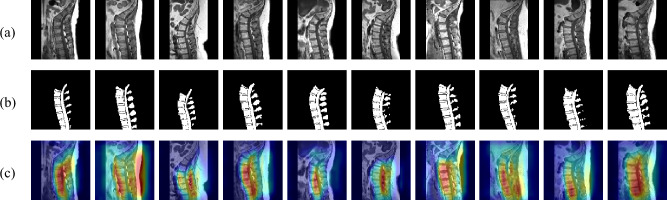
Test images with corresponding ground truth masks, and Grad-CAM visualizations for the proposed model. (**a**) Input Images. (**b**) Ground Truth Masks. (**c**) Proposed Model Grad-CAM.

Example sagittal spine MRI images with corresponding ground truth masks and Grad-CAM visualizations for the proposed model are presented in Fig. 6.

The heatmaps indicate that the proposed model consistently attends to spinal structures, particularly vertebral bodies, and IVDs, with high activation in critical regions. The strong correlation between high-activation areas and ground truth segmentation masks suggests that the proposed model effectively identifies key anatomical features. Additionally, the minimal activation in irrelevant regions highlights its robustness in distinguishing spine structures from surrounding tissues. These visualizations further confirm the proposed model’s capability to focus on clinically relevant regions, reinforcing its potential for real-world medical applications where interpretability and reliability are essential.

### Multiclass Segmentation Analysis

To extend its applicability beyond binary segmentation, the proposed model was adapted for multiclass segmentation, enabling the differentiation of three key spinal structures–vertebrae, IVDs, and the spinal canal–along with the background. This extension allows for a more detailed anatomical analysis, which is essential for clinical assessments and automated spine evaluation.

Segmenting vertebrae aids in structural analysis, spine curvature assessment, and the detection of abnormalities such as fractures or deformities. Identifying IVDs is crucial for diagnosing conditions like disc degeneration and herniation. Spinal canal segmentation plays a key role in detecting stenosis, compression, and other spinal cord-related pathologies. Finally, distinguishing the background ensures that the segmentation remains focused on relevant spinal structures without interference from surrounding regions.

Multiclass segmentation enhances the precision of computer-assisted diagnostic tools, supporting applications such as surgical planning, treatment evaluation, and automated pathology detection. By separately identifying spinal components, the model provides more granular insights into spinal health, reinforcing its significance in medical image analysis.

For multiclass segmentation, Categorical Cross-Entropy (CCE) was used instead of Binary Cross-Entropy (BCE) to handle multiple classes effectively. A total of twenty models were trained, including the baseline models–U-Net, ResUNet, Attention U-Net, TransUNet–and the proposed model, each evaluated using four different loss functions: CCE, Dice loss, Focal loss, and a combined CCE-Dice loss. Among these, the majority of models achieved their highest performance when trained with CCE-Dice loss, highlighting its effectiveness in optimizing segmentation accuracy. The performance metrics of all models trained with CCE-Dice loss are summarized in Table 9, providing a comparative evaluation of their segmentation effectiveness across the four classes.

**Table 9:** Comparative analysis of segmentation performance across different models trained with combined CCE-Dice loss for multiclass segmentation. The best results are indicated in bold.

Model	mIoU	Accuracy	Precision	Recall	F1-score
U-Net	0.8328	0.9710	0.9146	0.8983	0.9063
ResUNet	0.8229	0.9690	0.9000	0.9005	0.9001
Attention U-Net	0.8292	0.9700	0.8943	**0.9145**	0.9041
TransUNet	0.8301	0.9703	0.9030	0.9064	0.9047
**Proposed Model**	**0.8425**	**0.9726**	**0.9170**	0.9080	**0.9124**

Compared to the four baseline models, the proposed model achieved the highest mIoU (0.8425), accuracy (0.9726), precision (0.9170), and F1-score (0.9124), along with a strong recall (0.9080). While the recall was strong but not the highest, this can be attributed to the model’s focus on precise boundary delineation, which may have caused slight under-segmentation of some structures during multiclass prediction. These results confirm that the proposed architecture effectively extends its advantages from binary to multiclass segmentation, demonstrating strong adaptability in distinguishing multiple anatomical structures. However, as expected, overall performance declined compared to binary segmentation due to the increased complexity of differentiating four classes instead of two. The presence of additional class boundaries introduced higher inter-class similarity, making accurate segmentation more challenging.

The transition from binary to multiclass segmentation introduces several challenges. Class imbalance is a significant issue, as certain structures, such as the spinal canal, occupy a much smaller proportion of the image compared to vertebrae or background, potentially leading to biased predictions. Overlapping anatomical structures further complicate segmentation, particularly at the boundaries between vertebrae and IVDs, where distinctions are often ambiguous. Additionally, computational complexity rises with the increase in the number of classes, making learning more challenging and adding to the overall processing demands.

The combined CCE-Dice loss function helped mitigate these challenges by balancing per-class segmentation quality while optimizing for overlap-based accuracy. Despite these complexities, the proposed model consistently outperformed the baseline models, demonstrating its effectiveness in accurately segmenting spinal structures. Its ability to separately identify key spinal components makes it a valuable tool for advanced spine imaging analysis and automated diagnostic workflows in clinical applications.

**Fig. 7:**
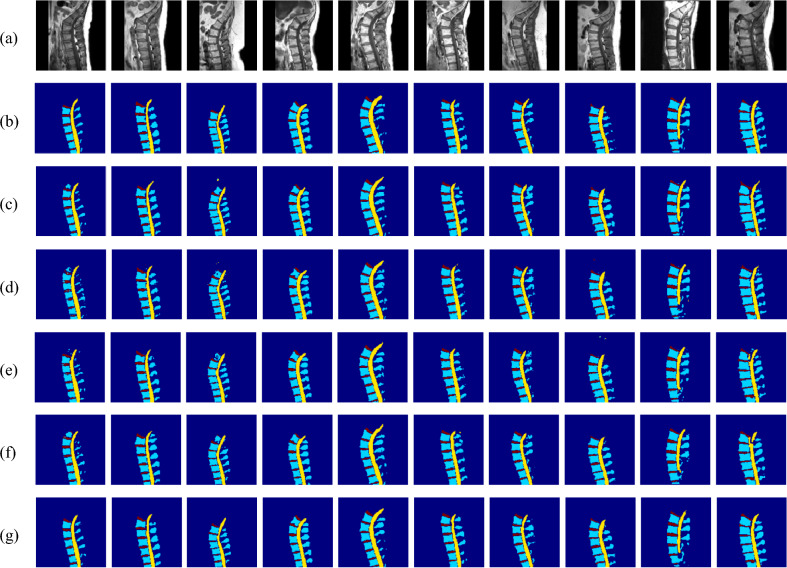
Test images with corresponding ground truth masks, multiclass predictions from four baseline models, and proposed model multiclass predictions.

Figure 7 presents qualitative comparisons of test images, ground truth masks, predictions from the four baseline models, and proposed model predictions for multiclass segmentation. The proposed model demonstrates more precise boundary delineation and fewer misclassifications than the baselines, effectively distinguishing vertebrae, IVDs, spinal canal, and background with improved segmentation accuracy.

## Conclusion

This research proposed an enhanced U-Net model incorporating an Inception module and a dual-output mechanism for improved lumbar spine segmentation in MRI. The model was trained on the SPIDER lumbar spine MRI dataset and demonstrated superior performance in segmenting vertebrae, IVDs, and the spinal canal. Segmentation was performed as a binary task, where spinal structures were considered as the foreground and all other regions as the background, enabling the model to learn a clear distinction between relevant anatomical structures and surrounding tissues.

The model was developed and evaluated specifically to improve binary segmentation accuracy and was compared against baseline U-Net and its enhanced variants–ResUNet, Attention U-Net, and TransUNet–demonstrating its effectiveness in capturing anatomical boundaries more precisely. To investigate the impact of training strategies, the model was trained using various loss functions, and comparative analysis identified Dice loss as the most effective, resulting in improved boundary delineation and higher segmentation accuracy.

The proposed model achieved an mIoU of 0.8974, accuracy of 0.9742, precision of 0.9417, recall of 0.9470, and an F1-score of 0.9444, surpassing all baseline models in segmentation performance. It effectively balanced computational efficiency with high segmentation accuracy, demonstrating its suitability for spinal MRI analysis.

To enhance interpretability, Grad-CAM visualization was used to highlight the regions influencing segmentation decisions, offering insights into how the model differentiates spinal structures. The study was later extended to four-class multiclass segmentation, enabling separate identification of vertebrae, IVDs, spinal canal, and the background, thereby improving anatomical precision and allowing the proposed model to outperform the baseline models in segmentation accuracy and boundary delineation.

Future work could explore segmenting each vertebra and each IVD individually to enable finer anatomical delineation, supporting more precise diagnosis and treatment planning. Expanding the dataset to include diverse imaging conditions, patient demographics, and spinal abnormalities could improve the model’s generalizability. Integrating multi-modal imaging data, such as CT scans or functional MRI, could further enhance robustness. Moreover, semi-supervised or self-supervised learning techniques, along with transformer-based architectures, could be explored to reduce dependency on large annotated datasets while improving segmentation quality.

## Data Availability

The dataset analyzed in the current study is available in the repository, https://spider.grand-challenge.org/data

## Declarations

### Code Availability Statement

The code used to implement the proposed model and perform the experiments will be made publicly available on GitHub upon publication of the manuscript.
